# DeepLocBox: Reliable Fingerprinting-Based Indoor Area Localization

**DOI:** 10.3390/s21062000

**Published:** 2021-03-12

**Authors:** Marius Laska, Jörg Blankenbach

**Affiliations:** Geodetic Institute and Chair for Computing in Civil Engineering & Geo Information Systems, RWTH Aachen University, Mies-van-der-Rohe-Str. 1, 52074 Aachen, Germany; blankenbach@gia.rwth-aachen.de

**Keywords:** indoor area localization, deep learning, fingerprinting, multi-building, multi-floor

## Abstract

Location-based services (LBS) have gained increasing importance in our everyday lives and serve as the foundation for many smartphone applications. Whereas Global Navigation Satellite Systems (GNSS) enable reliable position estimation outdoors, there does not exist any comparable gold standard for indoor localization yet. Wireless local area network (WLAN) fingerprinting is still a promising and widely adopted approach to indoor localization, since it does not rely on preinstalled hardware but uses the existing WLAN infrastructure typically present in buildings. The accuracy of the method is, however, limited due to unstable fingerprints, etc. Deep learning has recently gained attention in the field of indoor localization and is also utilized to increase the performance of fingerprinting-based approaches. Current solutions can be grouped into models that either estimate the exact position of the user (regression) or classify the area (pre-segmented floor plan) or a reference location. We propose a model, DeepLocBox (DLB), that offers reliable area localization in multi-building/multi-floor environments without the prerequisite of a pre-segmented floor plan. Instead, the model predicts a bounding box that contains the user’s position while minimizing the required prediction space (size of the box). We compare the performance of DLB with the standard approach of neural network-based position estimation and demonstrate that DLB achieves a gain in success probability by 9.48% on a self-collected dataset at RWTH Aachen University, Germany; by 5.48% for a dataset provided by Tampere University of Technology (TUT), Finland; and by 3.71% for the UJIIndoorLoc dataset collected at Jaume I University (UJI) campus, Spain.

## 1. Introduction

Driven by the wide adoption of smart devices, location-based services (LBS) are continuously gaining importance in our everyday lives. The user’s location can be determined by Global Navigation Satellite Systems (GNSSs) that offer reliable and accurate satellite position estimations. However, GNSSs do not sufficiently function inside buildings, since the signals are attenuated and scattered by building components. Although the potential usage of LBS within closed environments is estimated as high, the absence of a fully mature technological solution has restrained the growth of indoor LBS [1]. Therefore, there is an increased demand in developing alternative localization systems that can be applied indoors.

Existing solutions pursue different objectives. In monitor-based systems, the location of the user or entity is passively obtained with respect to some reference node [2], which can be used to develop context-aware systems that, for example, automatically regulate heating/cooling based on the presence or absence of people [3,4]. Especially, device-free localization has gained increasing attention in that regard. By analyzing a collected fingerprint from one or multiple access points at a certain fixed detection point, a target object can be accurately localized without being equipped with any additional device [5,6]. In device-based systems, the location is determined from a user-centric perspective by predominantly utilizing the smartphone as the localization device. This enables, e.g., navigation within complex environments [7,8,9]. Furthermore, it has the advantage that the user can determine his/her location without the requirement of data exchange with a centralized entity. Irrespective of the usage scenario, a wide range of different techniques and technologies are investigated in the field of indoor localization and are thoroughly discussed in [2,10,11,12].

Subsequently, we primarily focus on user-centric localization. Most of the existing device-based approaches can be seen as a compromise between the localization performance and the costs associated with localization systems that result, for example, from installation of the necessary infrastructure. By installing dedicated transmitters (e.g., Ultra-wideband (UWB)) within a building, sub-meter localization accuracy can be reached, which in return comes with a considerable amount of infrastructure planning and costs [13]. On the other hand, solutions that only reuse existing radio infrastructures within the building come at minimal cost but are limited with respect to the localization accuracy and reliability that they provide [2].

Fingerprinting-based localization approaches do not require additional hardware to be installed, which is the reason why they are still heavily investigated although they are naturally limited in the offered performance. The method builds on the fact that, depending on the location within a building, a different fingerprint of radio signals is observable. In the most widely applied implementation, the fingerprint consists of the vector of received signal strengths (RSS) to the observable wireless local area network (WLAN) access points (WLAN fingerprinting). In the so-called offline phase, those fingerprints are collected at certain reference points, such that the set of fingerprints labeled with the position of collection is obtained. The dataset can be utilized to model the relation between fingerprints and position with the goal of predicting the position of an unseen fingerprint during the localization phase (online phase).

This problem formulation is a natural fit for supervised machine learning, which is why the recent popularity of deep learning led to an increasing amount of publications that successfully apply deep learning for fingerprinting-based indoor localization [14,15,16,17,18]. RSS results from the superposition of multipath components and, as a consequence, fluctuates even at a static detection point [6], which limits the theoretically achievable localization performance. Therefore, approaches that trade expressiveness for reliability are becoming more popular [19,20,21]. By estimating a broader area or space instead of pinpointing the exact location of the user, the success rate of the prediction can be improved. An optimal space estimation model should provide a required success probability at the lowest possible average prediction space.

In this work, we propose a model (DeepLocBox) that offers reliable area localization in multi-building/multi-floor environments without the prerequisite of a pre-segmented floor plan. Instead, by using deep learning, it estimates an area (bounding box) large enough to contain the position of the user but as small as possible to maximize the knowledge gain. The model consists of a custom label encoding scheme, a dedicated output layer, and a custom loss function, whereas the neural network base can be interchanged. We demonstrate that applying the DLB head and loss function results in a considerable performance gain compared to standard regression head with mean-squared error (MSE) loss. Our data encoding scheme supports multi-building and multi-floor settings, such that a single model can be trained for the task of simultaneous building/floor classification and coordinate estimation.

We start by introducing related work in the field of device-based fingerprinting indoor localization in Section 2. Subsequently, we define a metric to quantify and compare the localization performance of different deep models with respect to their success probability and their expressiveness in Section 3. In Section 4, we introduce the DeepLocBox (DLB) model. In Section 5 DLB is evaluated on two public datasets and on a self-collected fingerprinting dataset. The results are presented and discussed for the single floor as well as for the multi-building/multi-floor setting. Finally, in Section 6, we conclude our results.

## 2. Related Work

### 2.1. Quantification of Localization Performance

According to ISO 5725-1:1994 (en) [22], *accuracy* is defined as the closeness of agreement between a test result and the accepted reference value. In the case of fingerprinting-based indoor localization, the test result is the position prediction of the model and the accepted reference value corresponds to the ground truth position where the fingerprint was collected. In the literature, accuracy is often reported as a statistic quantity determined on a dedicated test set of fingerprints that is unknown to the model. This can be, for example, the mean, median, or root mean square error (RMSE) between predicted and ground truth position. In ISO 5725-1:1994 (en) [22], *precision* is defined as the closeness in agreement between independent test results. It depends on the distribution of the error and can be reported by statistic measures such as the standard deviation. For evaluation of indoor localization systems, the precision of a model is often reported as a cumulative distribution function (CDF) [12,23]. In [24], precision is further defined as the success probability with respect to predefined accuracy. This can be seen as the percentage of correctly classified cases in space-based prediction systems [25]. For localization systems that predict one of few predefined classes (e.g., building, rooms, etc.), the classification accuracy can be reported as can as the confusion matrix and more sophisticated metrics that are built upon them [26].

In Section 3, we further address the accuracy and reliability of space-based estimation systems that do not necessarily require predefined classes. We develop a metric for the goal of reliable space estimation, which enables the comparison of existing deep models that vary in prediction output format.

### 2.2. Deep Learning for Fingerprinting

Deep learning has been successfully applied to boost the performance of fingerprinting-based indoor localization systems [15]. The simplest form of a deep neural network (DNN), a multi-layer perceptron (MLP), consists of multiple fully connected sequential layers. Each layer computes multiple linear combinations of its inputs and applies a nonlinear activation function, such as rectified linear unit (ReLU) [27]. The amount of linear combinations is identified as the number of hidden units. The parameters are called weights and are learned by the network. In the case of image classification, convolutional layers are applied to extract local features from the image. Several convolutional filters are moved across the image to generate so-called feature maps. Pooling layers can be applied to downsample the resulting feature maps. Popular choices for pooling operations are invariant to image rotation such as Maxpooling [27]. Several other network architectures are applied such as recurrent neural networks (RNN), in particular long short-term memory (LSTM), which utilizes their internal state to work on sequential data. In the domain of indoor localization, they can be applied on trajectory estimation [28,29] rather than independent absolute position estimation, which we focus on in this work.

The output of the models depends on the modeled problem. Either the exact point of location is estimated via regression or a multi-class classification problem is solved. In the latter case, classes are fixed reference points, grid cells, or predetermined areas of a segmented floor plan. Depending on the problem, a loss function determines the fit of the model. Popular choices are MSE for regression and categorical cross-entropy for multi-class classification [30]. Since the optimal choice of weights that minimizes the loss function cannot be determined analytically, iterative procedures are required. Gradient descent methods use the gradients of the loss with respect to each weight to slightly adapt the weights in the negative direction of the gradient at the current point [27]. The gradients are obtained via a computationally efficient algorithm called backpropagation. For a comprehensive introduction into deep learning, we refer to [27].

In the following an overview is given of recent work that applies deep learning to fingerprinting-based indoor localization grouped by the tackled problem.

- *Point estimation*: Xiao et al. [31] modeled the problem as regression task and applied a deep MLP model to estimate the position. Jaafar and Saab [32] realized point estimation using a MLP regression model after initial room classification. Using the data collected during walking along a predefined path, Sahar and Han [33] as well as Xu et al. [34], Elbes et al. [35], and Chen et al. [15] utilized LSTM with a regression output layer to predict the exact position. Ibrahim et al. [36] utilized a convolutional neural network (CNN) on RSS time-series data to estimate the coordinate on the lowest layer of their hierarchical prediction model (building and floor on higher levels). In a multi-task deep learning system, Lin et al. [37] utilized a MLP with regression output at the final stage of their architecture to estimate the position of the user. Wang et al. [38] utilized Angle of Arrival (AoA) images extracted from channel state information (CSI) as input to train a CNN network with regression output for point estimation. Li et al. [39] predicted the uncertainty of the fingerprint location estimation via an artificial neural network (ANN). They used the uncertainty to adapt the measurement noise in an extended Kalman filter that integrates the WLAN fingerprinting information.
- *Grid-based classification*: Li and Lei [40] utilized MLP for grid-based outdoor classification based on Long-Term Evolution (LTE) signals. Hsieh et al. [16] train a one-dimensional CNN using RSS and CSI to classify the correct grid cell of a rectangular room plane.
- *Reference point classification*: Mittal et al. [41] as well as Sharan and Hwang [42] utilized CNN to predict a unique reference point location modeled as classification problem. Li et al. [43] applied restricted Boltzmann machines (RBM) on CSI fingerprinting data to estimate a reference point location. Chen et al. [44] tackled device-free localization. They located a person within a room (determined the correct reference point) by applying CNN on CSI data. Using geomagnetic field data, Al-homayani and Mahoor [45] classified the reference point of users carrying a smartwatch. Rizk et al. [46] utilized cellular data for deep learning-based reference point classification. In the work by Shao et al. [47], magnetic and WLAN data were combined using a CNN.
- *Area classification*: Whereas [48] Liu et al. estimated the probability over predefined areas, Laska et al. [18] proposed a framework for adaptive indoor area localization using deep learning to classify the correct segment of a set of predefined segments. Njima et al. [49] constructed 3D input images that consist of the RSS data and the kurtosis values derived from the RSS data. Those are fed to a CNN that predicts the correct area/region of a pre-segmented floor plan.
- *Building/floor classification*: Kim et al. [50] proposed a deep model consisting of stacked auto-encoders (SAE) and a MLP for hierarchical classification of buildings and floors. Gu et al. [51] utilized a combination of SAE on WLAN fingerprints and additional sensor data for floor identification. Song et al. [52] determined buildings and floors by combining SAE and a one-dimensional CNN. Additionally, they equiped their model with the standard regression head to estimate the position given a classified floor.

### 2.3. Technologies Applied for Fingerprinting

It has been demonstrated that deep learning is a valuable tool for fingerprinting-based indoor localization. While the RSS of WLAN access points (AP) is predominantly used to establish fingerprints [33,35,41], alternative approaches have been presented. CSI of WLAN APs is increasingly used, since it contains richer multipath information [53]. This allows for improving the localization performance of deep models [16,38]. However, it requires special hardware in terms of a selected set of network interface cards (NIC). In the context of monitoring-based localization that works device-free, this does not display a major restriction, since CSI data is mostly read and processed on access points or fixed computing devices that function as detection points [54].

In user-centric localization systems, the user determines its location based on the received signals of the environment. Those are collected via a mobile device, which is typically a smartphone in the case of pedestrian localization. Current mobile operating systems, however, do not allow for accessing lower levels of the network stack, which prevents the extraction of CSI data. Therefore, there still exists a strong demand for algorithmic solutions that improve the reliability of RSS fingerprinting systems, which is a key challenge of this work.

## 3. Quantification of Localization Performance

In several scenarios, a localization system must provide a certain level of reliability, more precisely, it should provide a correct location prediction at a required *success rate*. This success rate is naturally defined for any space estimation model, where the prediction is considered correct if the ground truth position resides inside the predicted area. Besides the reliability of a space estimation model, its performance depends on the *expressiveness*. A model that provides the same reliability but utilizes a smaller average prediction space (higher precision) provides a larger knowledge gain to the user. Additionally, shape and compliance with the underlying floor plan can contribute to the expressiveness of the area localization model. In this study, we mainly focus on the size of the predicted shape that we state as the main contributor to knowledge gain of the model. Given a required level of reliability, the goal becomes finding the model that provides this reliability at the highest possible expressiveness.

Current deep models provide several estimation output formats. In order to compare the reliability of existing approaches, the output should be transformed into an equivalent space estimation such that the success rate and the utilized space can be measured and compared.

**Figure 1:**
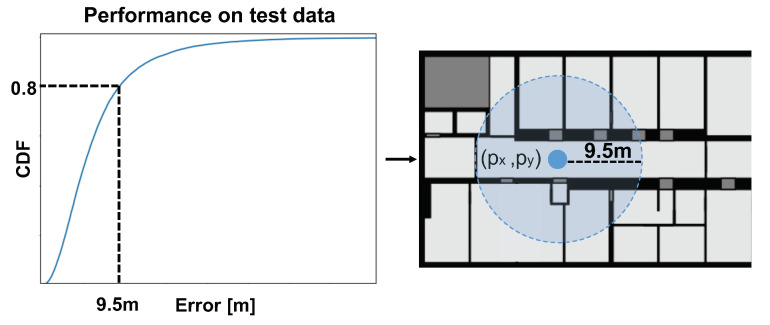
Transformation of the regression model output to space estimation via inspection of a cumulative distribution function (CDF).

Area classification models naturally provide a space estimation, such that its reliability can be quantified by the success rate of the classified area, while the required space can be computed as the average size of the predicted areas. The output of a point estimation model can be transformed into a space prediction by choosing the accuracy or tolerated error for a targeted prediction success rate. Since there is no knowledge about the direction of error, the estimated space can be modeled as a circle around the predicted point. The radius of the circle determines the success rate of the resulting space estimation model. It can be chosen by evaluating the CDF at the required level of reliability as illustrated in Figure 1. Assume that the estimated point of the model deviates less than 9.5 m in 80%. In that case, the radius of the circle can be chosen as 9.5 m, such that the transformed space estimation model will provide a success rate of 80%.

Let $FP=\{fp_{n}=\left(x_{n},l_{n}\right)\}$ for n=1,...,N, be the set of fingerprints, where $x_{n}$ is a D-dimensional fingerprint $x_{n}={\left(x_{1},...,x_{D}\right)}^{T}$ and $l_{n}={\left(l_{x},l_{y}\right)}^{T}$ is the two-dimensional position tag. We formally quantify both metrics for area classification models first and subsequently show how to transfer this to point estimation models.

### 3.1. Space Estimation

Let C be a model that predicts an area $a_{n}$ of any two-dimensional shape for an unknown fingerprint $x_{n}$ that belongs to the tagged location l. We say that C achieves a success rate of γ if

(1) $$\begin{array}{ccccc} & & \frac{\left|\{a_{n}|l_{n}\;\mathrm{inside}\;a_{n}\}\right|}{N} & \geq \gamma , & \forall n=1...N \end{array}$$

The smaller the predicted area, the higher the knowledge gain for the user. Therefore, the expressiveness of the model depends on the mean prediction space ϵ that is required to achieve the level of reliability, which is defined as

(2) $$\epsilon \left(C\right)=\frac{\sum_{n=1}^{N}area\left(a_{n}\right)}{N}\;,$$

where $area(a_{n})$ is defined as the surface area of $a_{n}$.

### 3.2. Point Estimation

Given a model R that estimates a position $\hat{p_{n}}={\left(\hat{p_{1}},\hat{p_{2}}\right)}^{T}$ that ideally corresponds to the target location $l_{n}$, we can transform it into a space estimation model that provides any chosen success rate. Let E(R) be the error vector of the model such that $E_{n}\left(R\right)=\left|\right|l_{n}-\hat{p_{n}}{\left|\right|}_{2}$. We can choose *r* such that

(3) $$\begin{array}{ccccc} & & \gamma & =\frac{\left|\{E_{n}|E_{n}\leq r\}\right|}{N}\;, & \forall n=1...N \end{array}$$

This means the error of the model is less than *r* in γ·100 percent of the cases. We can now predict the circle around $\hat{p_{n}}$ with radius r and can obtain an equivalent model that achieves a success rate of γ. For the transformed prediction, we obtain its required prediction space as

(4) $$\epsilon {\left(R\right)}_{\gamma }=\pi r^{2}\;.$$

## 4. DeepLocBox (DLB)

In the following, we introduce a new class of deep models for fingerprinting-based indoor localization that provides reliable space estimation without the prerequisite of a predetermined floor plan segmentation. Shapes are predicted individually for each fingerprint and, in contrast to area localization that constructs classes (shapes) based on a pre-segmented floor plan, can overlap for various predictions.

### 4.1. System Overview

**Figure 2:**
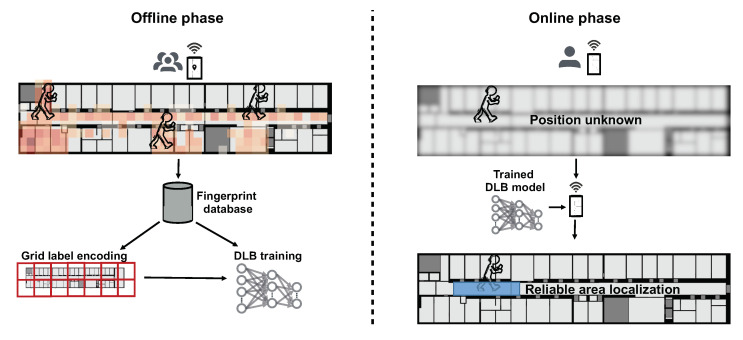
System overview.

The system overview is illustrated in Figure 2. Crowdsourced, labeled fingerprinting data are acquired during the offline phase and stored in a database. The floor plan is divided into large grid cells such that the model can learn to choose the right grid cell and can estimate the area of the user within the chosen grid cell’s coordinate system. In the online phase, a user can utilize the trained DLB model to reliably determine his/her current location area by the bounding box predicted by the model.

### 4.2. Model Description

The DLB model generates hierarchical predictions. It classifies a broad area, and within the area, it predicts a bounding box that contains the ground truth location. The broad areas consist of large square grid cells (chosen as 40 × 40 m), which should enable a high enough classification accuracy. Within each grid cell, a local coordinate system is used that has its origin at the center of the cell and its values range from −1 to 1. The prediction of the box within the cell consists of a 4-tuple $\left(c_{x},c_{y},w,h\right)$ with $c_{x},c_{y}\in \left[-1,1\right]$ representing the center of the bounding box and w,h∈[0,2] representing its width and height, respectively. Let $\left(t_{x},t_{y},t_{g}\right)$ be the target (ground truth location) encoded within the corresponding grid cell’s ($t_{g}$) coordinate system. The goal of the model is to predict the bounding box that contains $\left(t_{x},t_{y}\right)$ while minimizing the used area of the box. This is achieved via a custom loss function that consists of two components. The first component penalizes the distance of the predicted box center $\left(c_{x},c_{y}\right)$ to the target. The second component regulates the box dimensions by defining the bound that determines whether the box grows or shrinks. It depends on the ratio of the center error and the current dimensions of the box and can be parameterized via β. Formally, we define the loss of the box predictions as follows:

- *Center loss*: The center loss $E_{center}$ captures the deviation in the predicted center of the box from the ground truth point. $E_{center}$ is given as the squared distance:
  (5) $$E_{center}={\left(c_{x}-t_{x}\right)}^{2}+{\left(c_{y}-t_{y}\right)}^{2}\;.$$
- *Size loss*: The size loss regulates the box dimensions. We define it as
  (6) $$E_{size}={\left(|c_{x}-t_{x}|-w/\beta \right)}^{2}+{\left(|c_{y}-t_{y}|-h/\beta \right)}^{2}\;.$$

The final box loss function is given as the sum of the two loss components:

(7) $$E_{box}=E_{center}+E_{size}\;.$$

**Figure 3:**
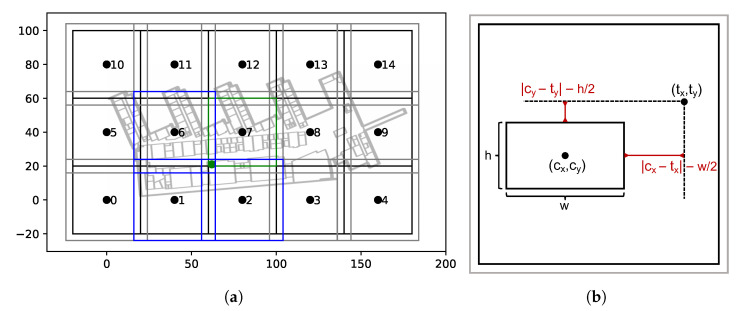
Illustration of the label encoding (**a**) and the encoding within each grid cell (**b**) depicted with the components of the loss function.

Note that, when setting β=2, $E_{size}$ corresponds to the squared distance from the boundary of the box to the target location, as depicted in Figure 3b.

The hierarchical prediction is performed end-to-end, meaning that a single execution of the model classifies the grid cell and predicts the bounding box within the chosen cell. This is realized by modeling the output as vector of length O=5*G:

(8) $$\begin{array}{cc} output= & (c_{x}^{\left(1\right)},c_{y}^{\left(1\right)},w^{\left(1\right)},h^{\left(1\right)},g^{\left(1\right)},...,\\ \; & c_{x}^{\left(G\right)},c_{y}^{\left(G\right)},w^{\left(G\right)},h^{\left(G\right)},g^{\left(G\right)})\;, \end{array}$$

where *G* is the number of grid cells. Each fifth entry $g^{\left(i\right)}$ of the output corresponds to the confidence of the model that the target is within the ith cell. The largest $g^{\left(i\right)}$ determines the chosen cell, and the corresponding box prediction $\left(c_{x}^{\left(i\right)},c_{y}^{\left(i\right)},w^{\left(i\right)},h^{\left(i\right)}\right)$ is used for computation of the box loss (Equation (7)). Let ${𝟙}_{i}$ be 1 if $i=t_{g}$ and 0 otherwise; furthermore, let $j=argmax\{g^{\left(1\right)},...,g^{\left(G\right)}\}$.

We define the composed loss function consisting of grid cell classification and box prediction losses as follows:

(9) $$\begin{array}{cc} L= & \alpha \;\cdot \;-\sum_{i=1}^{G}{𝟙}_{i}\;\cdot \;log\left(g_{i}\right)\\ \; & +sum\{{\left(c^{\left(j\right)}-t\right)}^{2}\\ \; & +{\left(|c^{\left(j\right)}-t\left|-d^{\left(j\right)}/\beta \right)\right)}^{2}\} \end{array}$$

with $d^{\left(j\right)}={\left(w^{\left(j\right)},h^{\left(j\right)}\right)}^{T}$, $c^{\left(j\right)}={\left(c_{x}^{\left(j\right)},c_{y}^{\left(j\right)}\right)}^{T}$, and $t={\left(t_{x},t_{y}\right)}^{T}$. The constant α is meant to balance the grid cell classification loss with the box loss. The upper bound of the box loss is ${\left(c_{x}-t_{x}\right)}^{2}+\left(\right|c_{x}-t_{x}{\left|-w/\beta \right)}^{2}\leq 4+4<10$ in each direction, such that we choose α=20 as our scaling parameter.

### 4.3. Data Encoding and Label Augmentation

In order to encode the data for the introduced model, the floor plan is divided into grid cells of fixed size (40 × 40 m) with an added padding zone around them. The encoding is illustrated in Figure 3a. Each location $\left(l_{x},l_{y}\right)$ is assigned to the closest grid cell with respect to center distance. The corresponding $\left(t_{x},t_{y}\right)$ is obtained by linearly transforming the original label into the local coordinate system of the grid cell with an origin at the center of the cell and the boundary (end of padding zone) at 1 and −1, respectively. The padding zone enables data augmentation. In contrast to classical augmentation known from image classification or explicitly adopted for fingerprinting [55], not the input data but the labels are augmented, which are capable of benefitting model performance as shown in [56]. In our setting, labels that lie within the padding zone of other grid cells can additionally be encoded within those coordinate systems. Especially for the joint task of grid cell classification, the augmentation scheme can reduce the regression error for misclassified grid cells. The encoding is exemplarily visualized in Figure 3a. The black squares depict the grid cells (40 × 40 m) with an added padding zone (grey) of 4 m per dimension. The green point represents a label $\left(l_{x},l_{y}\right)=\left(62,21\right)$, with the green cell (no. 7) being the closest one. For the green box, we end up with an encoding of $\left(t_{x},t_{y},t_{g}\right)=\left(-0.75,-0.79,7\right)$. Additionally, the label lies in the padding zone of the cells 1, 2, and 6. Analogously, we can obtain augmented encodings with respect to those grid cell origins, such that we end up with a total of 4 label encodings for the example fingerprint.

The model also supports scalable multi-building and multi-floor localization. This is realized by a flat encoding using the introduced grid-cell encoding for each building/floor combination separately. The resulting grid cell IDs can be explicitly mapped to a grid cell of the corresponding floor of the building. Let Ω be the index of a building floor combination (sorted by building ID and floor ID ascending). Furthermore, let $Enc^{\left(\Omega \right)}:{\left(l_{x},l_{y}\right)}^{\left(\Omega \right)}\to \left(t_{x}^{\left(\Omega \right)},t_{y}^{\left(\Omega \right)},t_{g}^{\left(\Omega \right)}\right)$ be the mapping introduced for building/floor combination Ω. Let $G^{\left(\Omega \right)}$ be the number of grid cells of $Enc^{\left(\Omega \right)}$. We can then define the multi-building and multi-floor encoding as $Enc:{\left(l_{x},l_{y}\right)}^{\left(\Omega \right)}\to \left(t_{x}^{\left(\Omega \right)},t_{y}^{\left(\Omega \right)},t_{g}^{\left(\Omega \right)}+\sum_{i=1}^{\Omega }G^{\left(i\right)}\right)$.

### 4.4. Derivatives of Loss Function

In the following, we derive the δ values of the loss function with respect to the output layer of the network. Those values can then be utilized during the backpropagation algorithm to obtain partial derivatives of the loss function with respect to each weight of the network to iteratively adjust the network weights. Given a loss function *E*, the goal is to obtain its derivatives with respect to the weights of each layer. *E* depends on a specific weight $w_{ji}$ only via the summed input $a_{j}$ to unit *j*, such that the chain rule for partial derivates can be applied [30]:

(10) $$\frac{\partial E}{\partial w_{ji}}=\frac{\partial E}{\partial a_{j}}\frac{\partial a_{j}}{\partial w_{ji}}\;.$$

Let $\delta_{j}$ be defined as $\delta_{j}=\partial E/\partial a_{j}$. Since $a_{j}=\sum_{i}w_{ji}z_{i}$, we see that $\partial a_{j}/w_{ji}=z_{i}$. Again, we can apply the chain rule to obtain the backpropagation formula that yields the δ of the previous layer as $\delta_{j}=h^{\prime }\left(a_{j}\right)=\sum_{k}w_{kj}\delta_{k}$, where k=1...K is the index of the output units and $j=1...H^{\left(L-1\right)}$ is the index over the units of the previous layer. Thus, it only remains to evaluate the δ for the output layer and to propagate it back to obtain the δ values of the previous layers. If we have stored the corresponding $z^{\left(i\right)}$ values (outputs of intermediate layers), we can then obtain the partial derivatives of *E* with respect to each specific weight, as $\partial E/\partial w_{ji}$ [30]. The process of obtaining the δ values is derived for the proposed box loss equations in the following. Recall that the loss is composed of two individual sub-losses that we can discuss individually, since they are additively combined.

- *Center loss*: $E_{center}$ is independent of the width and the height of the predicted box; therefore, it holds that
  (11) $$\begin{array}{cc} \delta_{3}^{L} & =\frac{\partial E_{center}}{\partial a_{3}}=\frac{\partial E_{center}}{\partial w}=0\;, \end{array}$$
  (12) $$\begin{array}{cc} \delta_{4}^{L} & =\frac{\partial E_{center}}{\partial a_{4}}=\frac{\partial E_{center}}{\partial h}=0\;. \end{array}$$
  The partial derivatives with respect to the center are given as
  (13) $$\delta_{1}^{L}=\frac{\partial E_{center}}{\partial c_{x}}=2\left(c_{x}-t_{x}\right)$$
  and analogously
  (14) $$\delta_{2}^{L}=\frac{\partial E_{center}}{\partial c_{y}}=2\left(c_{y}-t_{y}\right)\;.$$
- *Size loss*: The delta values of $E_{size}$ are given as
  (15) $$\begin{array}{ll} \frac{\partial E_{size}}{\partial a_{1}} & =\frac{\partial \;(|c_{x}-t_{x}{\left|-w/\beta \right)}^{2}}{\partial c_{x}}\\ & \begin{array}{l} =\left\{\begin{array}{cc} 2|c_{x}-t_{x}|-\frac{2}{\beta }w, & \mathrm{if}\;c_{x}>t_{x}\\ -2|c_{x}-t_{x}|+\frac{2}{\beta }w, & \mathrm{if}\;c_{x}<t_{x}\\ \mathrm{undefined}, & c_{x}=t_{x} \end{array}\right. \end{array} \end{array}$$
  (16) $$\begin{array}{ll} \frac{\partial E_{size}}{\partial a_{2}} & =\frac{\partial \;(|c_{y}-t_{y}{\left|-h/\beta \right)}^{2}}{\partial c_{y}}\\ & \begin{array}{l} =\left\{\begin{array}{cc} 2|c_{y}-t_{y}|-\frac{2}{\beta }h, & \mathrm{if}\;c_{y}>t_{y}\\ -2|c_{y}-t_{y}|+\frac{2}{\beta }h, & \mathrm{if}\;c_{y}<t_{y}\\ \mathrm{undefined}, & c_{x}=t_{x} \end{array}\right. \end{array} \end{array}$$
  (17) $$\begin{array}{cc} \frac{\partial E_{size}}{\partial a_{3}} & =\frac{\partial \;(|c_{x}-t_{x}{\left|-w/\beta \right)}^{2}}{\partial w}\\ \; & =-\frac{2}{\beta }\left(\right|c_{x}-t_{x}\left|-\frac{1}{\beta }w\right) \end{array}$$
  (18) $$\begin{array}{cc} \frac{\partial E_{size}}{\partial a_{4}} & =\frac{\partial \;(|c_{y}-t_{y}{\left|-h/\beta \right)}^{2}}{\partial h}\\ \; & =-\frac{2}{\beta }\left(\right|c_{y}-t_{y}\left|-\frac{1}{\beta }h\right) \end{array}$$

With the introduced scaling factors, the final delta values for the output layer are exemplarily given as

(19) $$\delta^{L}=\left(\begin{array}{cc} & 4|c_{x}-t_{x}|-\frac{2}{\beta }w\\ & -4|c_{y}-t_{y}|+\frac{2}{\beta }h\\ \; & -\frac{2}{\beta }\left(\right|c_{x}-t_{x}\left|-\frac{1}{\beta }w\right)\\ & -\frac{2}{\beta }\left(\right|c_{y}-t_{y}\left|-\frac{1}{\beta }h\right) \end{array}\right)$$

in the case that the ground truth point lies left from the horizontal box boundary and above the vertical box boundary. Other cases can be derived via the equations above. In the presented case, this causes a center move to left (weight update in the negative direction of loss) by a factor of $4|c_{x}-t_{x}|-\frac{2}{\beta }w$ and a center move downwards by $-4|c_{y}-t_{y}|+\frac{2}{\beta }h$.

The sign of $\delta_{3,4}^{L}$ determines whether the box size increases or decreases. Via adjusting the β parameter, the bound can be shifted, which results in higher accuracy and larger box sizes and vice versa. In the case where $\delta_{3,4}^{L}$ becomes negative, the box grows, which is given in the x-direction if $\frac{2}{\beta^{2}}<\frac{2}{\beta }\left|c_{x}-t_{x}\right|$. More precisely, it must hold that

(20) $$w<\beta |c_{x}-t_{x}|\;.$$

This means that the box width must not be greater than β times the error in the x-direction for the box width to grow.

## 5. Evaluation

We initially evaluate DLB on a self-collected dataset and subsequently reinforce the validity by an evaluation on two public independent datasets for fingerprinting-based indoor localization.

First, the single floor setting is studied followed by an analysis of multi-building/multi-floor localization. We utilize two base architectures (DNN [31] and 2D-CNN [42]). In the first case, we equip them with a two-dimensional regression output layer and mean squared error (MSE) loss. We refer to them as point estimation models in the following. In the other case, we utilize our DLB neural network head and refer to them as DLB models in the following. We artificially transform the output of the point estimation models to a space estimation by predicting a circle with fixed radius such that the model provides an adjustable success rate that depends on the radius (see Section 3). We investigate the achieved success rate of both model types with respect to the required average prediction space. An optimal model should provide a high success rate while requiring as little prediction space as possible. In that regards, we show that the proposed DLB models significantly outperform the space estimation resulting from the point estimation models. Furthermore, we show that the proposed label augmentation strategy (see Section 4.3) further benefits the performance of the DLB models.

### 5.1. Model Architecture and Preprocessing

The model parameters are depicted in Table 1 and Table 2, where square brackets indicate that multiple configurations were chosen. Hidden layers are abbreviated as HL, and hidden units are abbreviated as HU. The model weights are learned using the Adam optimizer.

The input layers of the networks receive the RSS scaled to [0,1] of all access points. Missing values are replaced with a value of −110 dB before scaling. The performance is validated on a separate testing dataset. Details regarding the splitting strategy are reported in the subsequent section for each dataset individually. The training of neural networks is non-deterministic, such that training two models with the same parameters might result in different model weights and, as a consequence, in different outputs and performances. To report statistically significant results, we train each model 10 times and average the resulting performance.

### 5.2. Datasets

In the following, the datasets are introduced that serve as a basis for the evaluation.

#### 5.2.1. Dataset Collected in RWTH Aachen University

**Figure 4:**
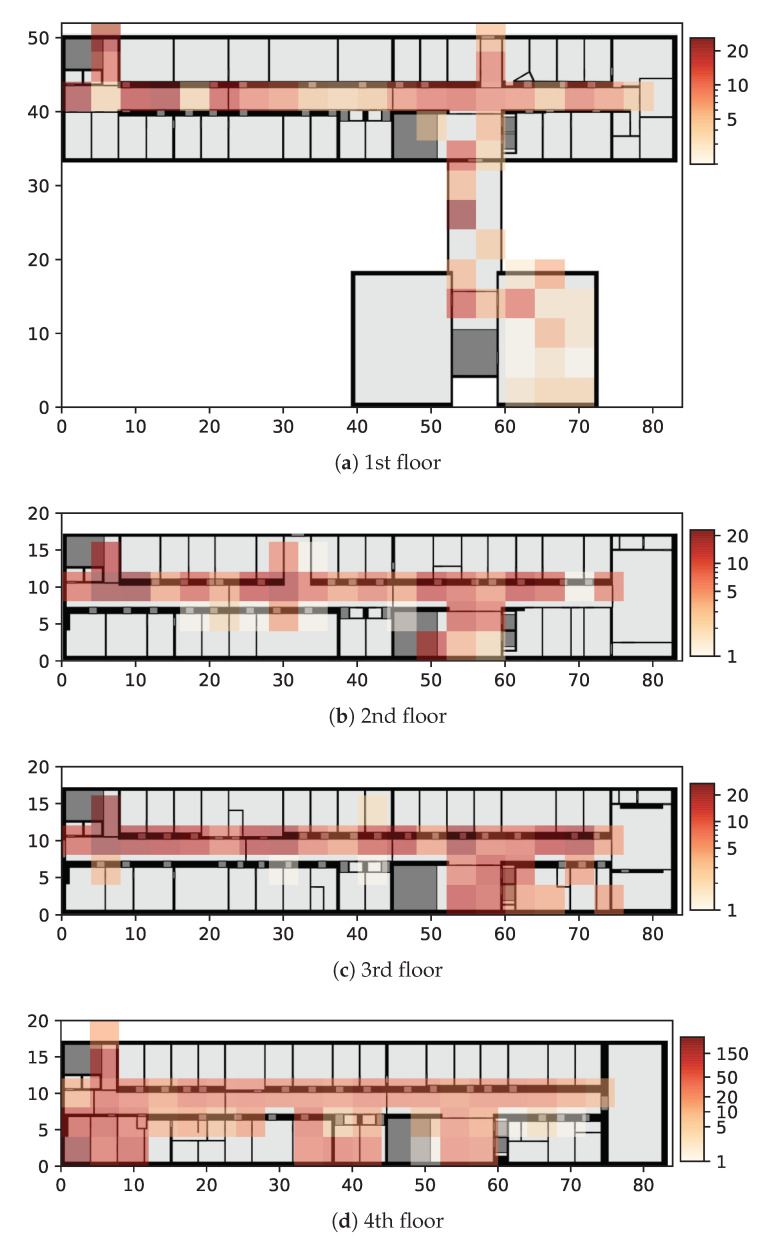
Illustration of the data distribution of the RWTH Aachen dataset. The heatmap visualizes the amount of labeled data per 4 × 4 m grid cell.

The dataset was collected at a building of RWTH Aachen University, Germany. The building consists of 7 floors, of which the first 4 are covered in the dataset. The data were collected by 7 smartphones over the period from December 2018 to August 2020 with multiple peak collection phases. During the course of collection, the WLAN infrastructure remained mostly stable. Data collection and tagging were realized via a smartphone application that allowed us to manually select the current sampling location. In total, more than 3000 fingerprints were collected, with the highest sampling density (1866 samples) being on the 4th floor. The data distribution is visualized in Figure 4. We utilized the 4th floor as a subset for the single floor evaluation and randomly split the data into 5 different cross validation folds.

#### 5.2.2. UJIIndoorLoc Dataset

**Figure 5:**
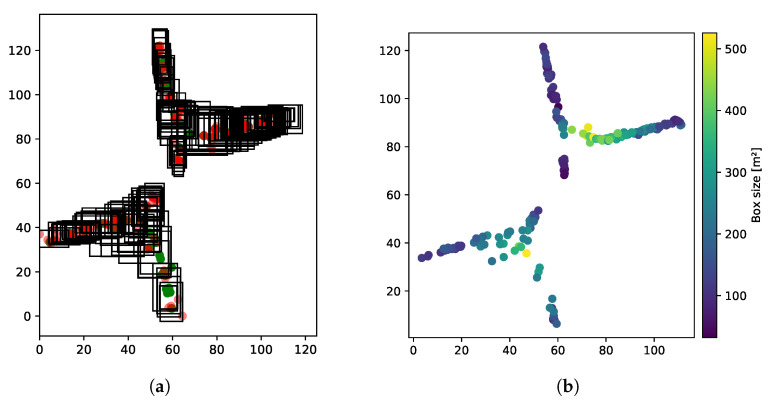
Illustration of DeepLocBox (DLB) output on the Jaume I University (UJI) dataset. The predicted boxes of the DLB model for floor 0 of building 0 are presented in (**a**) of the DNN–DLB (1 HL) and β=20. The sizes of the boxes are visualized in (**b**).

The UJIIndoorLoc dataset [57] contains more than 20,000 labeled fingerprints that are distributed over three multi-level buildings located at Jaume I University (UJI). The dataset was published with a predetermined split into training and testing data (collected 4 month later). The data were collected by more than 20 users using 25 different models of mobile devices [57]. We utilized building 0 as a subset for our validation and adopted the proposed split into training and testing data. For illustration purposes, the output of one DLB model is visualized in Figure 5. In Figure 5a, the green dots represent the training point locations and the red dots mark the testing reference points. The black boxes represent the bounding boxes predicted by the DLB model. Figure 5b shows the size of the predicted boxes.

#### 5.2.3. Dataset Collected in Tampere, Finland

**Figure 6:**
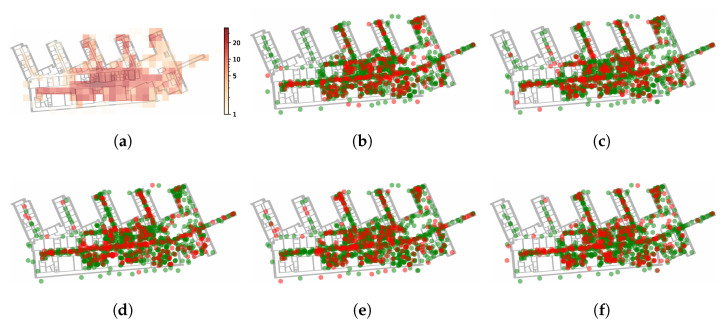
Illustration of the data distribution of the Tampere dataset. The heatmap (**a**) visualizes the amount of labeled data per 6 × 6 m grid cell. (**b**–**f**) depict the 5 splits into train (green) and test data (red) used during evaluation.

The dataset [58] collected in a university building in Tampere, Finland, consists of 4648 fingerprints recorded by 21 devices. The fingerprints were distributed over five floors, whereas the 1st floor had the highest sample density and was chosen for evaluation in the single floor setting. The dataset was published with a predetermined train/test split consisting of 20% training and 80% testing data. After inspecting the distribution of training data of the 1st floor, we noted that only a single area of the split contains training data while the rest was labeled as testing data. Such a split was impractical for the single floor setting, which was why we applied 5-fold cross validation instead. The splits are visualized in Figure 6.

### 5.3. Performance Analysis

#### 5.3.1. Single Building/Floor Positioning

**Figure 7:**
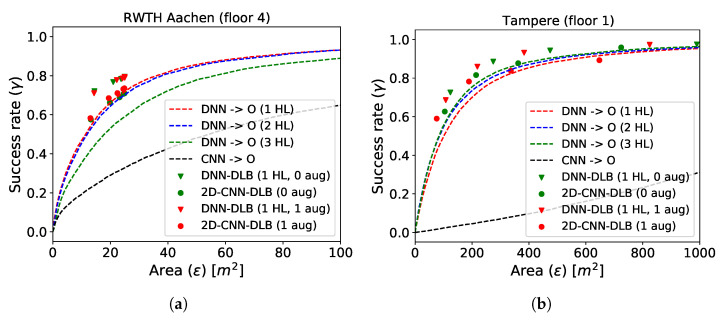
Performance comparison of DLB and DNN models on datasets collected at RWTH (floor 4) (**a**) and in Tampere (floor 1) (**b**).

**Figure 8:**
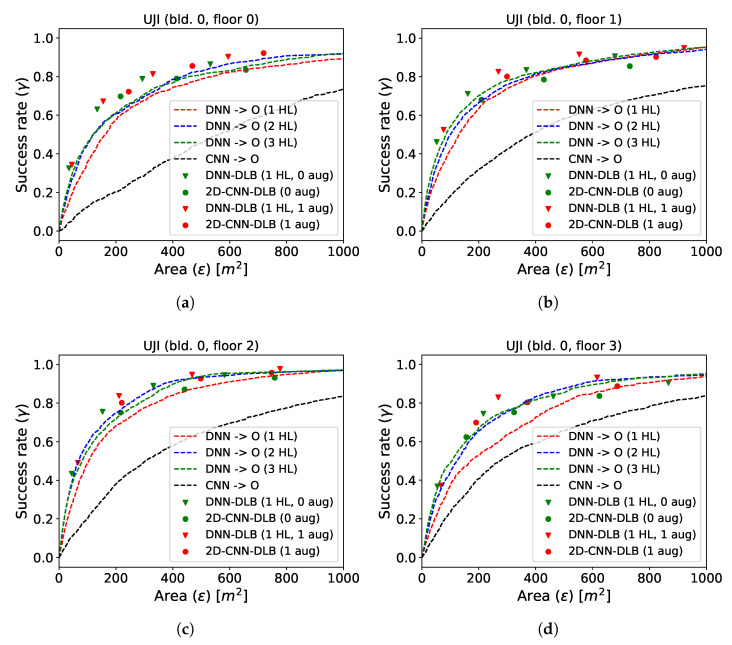
Performance comparison of the DLB and DNN models on the UJI dataset. (**a**–**d**) show the floors 0–3.

In the upcoming section, we focus on models trained for single building/floor settings. The resulting performance on the test data is presented in Figure 7 for datasets collected at RWTH (a) and in Tampere (b) and in Figure 8 for the UJI dataset.

The dashed curves show the performance of the space estimation models by varying the size of circle around the predicted point. The DNN space estimation models with 2 hidden layers perform best at RWTH, whereas 3 hidden layers are optimal at Tampere and UJI. Increasing the depth of the model further did not result in an additionally increased performance and is thus omitted in the plot. The DLB models are represented by the triangles. Since they predict boxes, their performance evaluation on the same testing data results in a fixed success rate and in an on-average utilized prediction space. The red triangles represent the DLB models that were supplied with the additionally augmented data during training, while the green triangles show the performance of the DLB models using only the base training data. Only models with 1 hidden layer are depicted, as the performance did not increase with model depth from that point. The parameter β has been introduced in Equation (6) as a way to alter the bound that determines whether box size is increased or decreased during training. In Section 4.4, it has been established that the box will increase as long as the current box width is not greater than β times the current error in the x-direction. The same holds independently for the ratio between box height and error in the y-direction. To obtain various success rates of the DLB models, we varied the β parameter (β∈{5,7.5,10,15} for RWTH and Tampere and β∈{5,10,15,20} for UJI). The target area of reliable space estimation models that use as little space as possible resides in the upper left corner of Figure 7 and Figure 8 (high success rate and minimal prediction space). It can be seen that, for all test sites, the DNN–DLB models outperform the DNN space estimation models by a considerable margin. The 2D-CNN space estimation models do not achieve satisfactory results. However, when equipping them with the DLB head, their performance can be clearly improved. The exact performance gain with respect to success rate and expressiveness can be obtained by measuring the vertical or horizontal distances from the triangles (DLB models) to the curves (space estimation models). The results are presented in Table 3.

*SUCC-gain* corresponds to the average vertical distance between the DNN–DLB models and the best performing DNN. It is reported for both the model trained on the base training data (no aug.) and for those trained on the augmented training data (aug.). Additionally, we list the percentage of additional training data that results from the proposed data augmentation scheme for each test site. The *Size-gain* corresponds to the average horizontal distance between the DLB models and the best performing DNN. We can conclude that the DNN–DLB models trained on the base data achieve a gain in success rate by 9.46%, 5.48%, and 3.71% for the various test sites when using the same prediction space. When comparing the utilized space for models that have the same success rate, the DNN–DLB models require 9.97 m${}^{2}$, 179.23 m${}^{2}$, and 12.56 m${}^{2}$ less prediction space test site-wise. When using the augmented data, the performance can be slightly increased to a success rate gain of 9.08%, 6.66%, and 3.86% and the prediction space at a fixed success rate decreases by 9.54 m${}^{2}$, 207.33 m${}^{2}$, and 103.74 m${}^{2}$.

We further analyze the difference between the center of the predicted boxes and the ground truth point, which should ideally be as close as possible, and compare it with the error of the DNN point estimation model. The results are reported for the best performing model of each category in Table 4.

At RWTH Aachen, the DLB model on the non-augmented data outperforms the other models and achieves mean and median errors of 2.04 m and 1.25 m, respectively. For the data collected in Tampere, the DNN–DLB with 1 hidden layer trained on the augmented training data achieved the best results. It has the lowest mean and median errors of 5.33 m and 4.09 m, respectively. For the UJI dataset, the same DLB model configuration outperformed all other models with reported mean and median errors of 6.31 m and 4.75 m, respectively. While the DLB models have a lower center error for all reported quantiles, the maximum error is considerably higher at UJI and slightly higher at RWTH and Tampere. Thus, it can be noted that the overall center error is lower and that the mass of its distribution is shifted towards the lower end (even the 75% quantile is lower). However, the DLB produce a few outliers, which can be attributed to classifying the wrong grid cell. The 2D-CNN models achieve weak results with respect to positioning error. However, when equipped with the DLB head, the center error resides in a comparable range to the other models. At the UJI test site, the 2D-CNN–DLB even has lower mean and median center errors than the best DNN (3 HL) model.

#### 5.3.2. Multi-Building/Multi-Floor Positioning

The proposed DLB neural network head is also suitable for scalable multi-building/ multi-floor positioning. This can be achieved by following the flat encoding strategy proposed in Section 4.3. For the RWTH dataset, we train a multi-floor model with a grid cell size of 5 × 5 m, 1 hidden layer with 512 hidden units and a dropout layer (*p* = 0.5). We obtain a floor success rate of 99.5% with mean and median errors of 2.06 m and 1.22 m. The reported performance is very close to the single floor accuracy and demonstrates that we can utilize a single multi-floor model without sacrificing any localization accuracy.

In addition, we train a single DLB model for the UJI dataset with a one hidden layer consisting of 384 hidden units and a dropout layer (*p* = 0.4). At the multi-building/multi-floor positioning task, our model outperforms the state-of-the-art scalable neural network approach [50] with respect to floor detection rate and positioning error. We also put the results in comparison to hierarchical approaches (multi-model) both of the International Conference on Indoor Positioning and Indoor Navigation (IPIN) 2015 competition [59] and a recent multi-model approach [52]. Hierarchical models in general achieve better performance but do not scale well, since separate models for the objective of building classification, floor classification for each building, and position estimation for each floor of each building are required. Especially in median positioning error, our single DNN–DLB model achieves results that can keep up with the best multi-model results. The performance comparison is displayed in Table 5. We report the best and average performances of 10 runs with varying speeds.

### 5.4. Discussion

During the evaluation, it has been shown that the proposed DLB models estimate the user’s area with a higher success probability while using less average prediction space. The DLB model is meant to be applied in combination with existing deep architectures by only changing its output layer, the utilized loss function, and the data encoding. This has been demonstrated using a standard MLP network equipped with the DLB head and trained with the DLB loss and comparing its performance to the same MLP network with standard regression output layer and MSE loss function. The same approach was followed for a 2D-CNN network. The neural network base architectures were identical, as reported in Table 1 and Table 2. Furthermore, the networks were trained on the exact same data and tested on the exact same data. Still, the models equipped with DLB achieved a significant increase in success rate and decrease in utilized prediction space, respectively. One the one hand, this can be attributed to the lower box center error. On the other hand, it might be the ability of the model to directly predict the box size. However, this claim has to be discussed more thoroughly.

#### 5.4.1. Correlation between Box Size and Center Error

The objective of DLB is to choose the box size based on certainty. As a consequence, when evaluating the model on unseen data, the box size should be larger for predictions where the box center differs to a greater extent. This is a difficult task, since the model has to guess its deviation from the ground truth position. To check the DLB model capability in that regard, we investigated the correlation between the center error and size of the boxes predicted for unseen fingerprints. Our analysis was conducted on a subset of the DNN–DLB models trained on floor 0 of building 0 of the UJI dataset. We fit multiple univariate linear regression models to show two hypotheses:

(1) A significant positive correlation between the box size and the center error of the predicted boxes exists.
(2) The correlation between error and box size is larger for the same components as opposed to the opposite components.

To simplify the notations, we introduced $e=||c-{t||}_{2}$, $e_{x}=\left|c_{x}-t_{x}\right|$ and $e_{y}=\left|c_{y}-t_{y}\right|$. The results are presented in Table 6.

In order to accept the first hypothesis, we have to investigate the significance of the coefficient estimates and the general fit of the model. We have to check whether the null hypothesis, which states that there is no relationship between *e* and w·h, can be rejected. By applying t-statistic, we obtain the *p*-values. A small *p*-value indicates that it is unlikely to observe such a substantial association between the predictor and the response due to chance [60]. The results show very low *p*-values of at most 2.64 × 10^−14^ for the $\beta_{1}$ coefficients, such that we can safely reject the null hypothesis.

The obtained $R^{2}$ values lie between 0.1 and 0.2, which means that roughly 10–20% of the variation in w·h is explained by the model. The box size might depend on other factors, which is why the linear regression with the error as the single predictor is not expected to give a perfect fit.

To investigate the second hypothesis, we correlated the box size and the center error component-wise. We note that the correlation is higher and more significant for the same component. Informally, this means that the model will choose a wider box if it expects the error to be larger in the x-direction but a smaller (height) box if the error is likely to be smaller in the y-direction.

**Figure 9:**
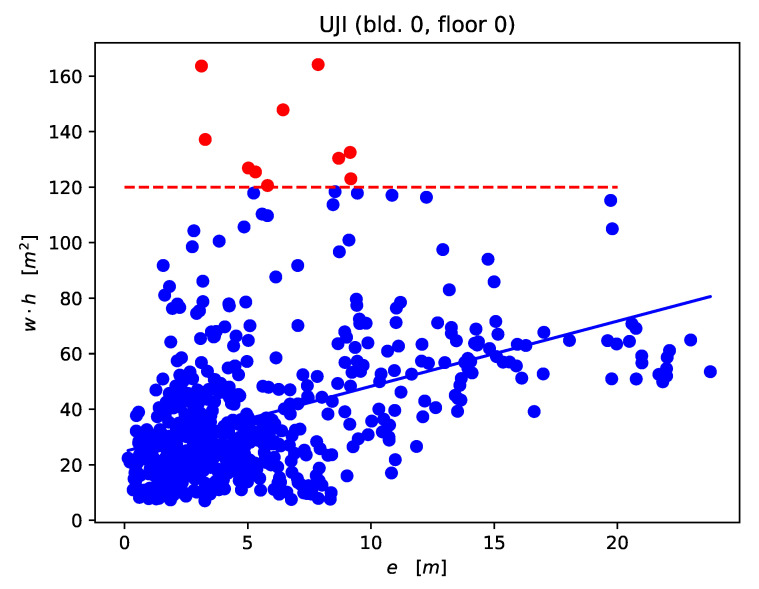
Correlation between box size (w·h) and center error (e) of model DNN–DLB (1 HL, 0 aug, β=5). Red points mark outlier predictions where the box size is larger than 120 m${}^{2}$ (1.6% of the predictions).

The correlation between center error (*e*) and box size (w·h) is visualized in Figure 9. A scatter plot is shown together with the fitted regression line for the model DLB–DNN (1 HL, 0 aug). When considering the red points (above 120 m${}^{2}$) as noise, we discard a total of 1.6% of the predictions and obtain a $R^{2}$ value of 0.22. Setting the threshold bound at 75 m${}^{2}$ (7% discarded) yields a $R^{2}$ value of 0.3.

We conclude that the size of the boxes predicted by the DLB models for unseen fingerprints correlates with the deviation of the box center from the ground truth point. The correlation is significant (by analysis of the *p*-value); however, there still exists a high variance, which causes the $R^{2}$ values to be relatively low. This prevents interpretation of the box size as a direct certainty measure on an individual prediction basis. However, due to the shown correlation, the DLB models on average require less prediction space to guarantee the same success rate, as shown in Section 5.3.1. In particular, the DLB models that choose the box size on an individual prediction basis outperform the same models that assume a constant prediction uncertainty modeled as a circle around the estimated position. One objective of future work should be to increase the correlation between error and box size that is learnt by the model such that we can safely interpret the box size as a measure of certainty.

#### 5.4.2. Multi-Building/Multi-Floor Performance

We assessed the ability to apply DLB for scalable multi-building/multi-floor localization. Our approach outperformed the state-of-the-art single DNN for multi-building/multi-floor localization with respect to floor detection rate and positioning error. Since there is no CDF reported for the reference models, we are only able to compare the center error of the box predicted by the DNN–DLB with the point estimation error of the other models. However, models equipped with DLB are meant to achieve a higher success rate (ground truth point within box) while using fewer prediction space as models such as [50,52]. This was demonstrated in the single-floor evaluation in Section 5.3.1 and discussed in Section 5.4.1.

#### 5.4.3. Limitations

One limitation of DLB is that it is tied to predicting axis-aligned rectangular boxes. This works especially well for classic building structures but has its limits for non-axis-aligned building shapes, such as the ones of the test sites. A potential improvement could be to allow more complex polygon predictions.

## 6. Conclusions

Fingerprinting is still an attractive approach for device-based—especially smartphone-based—indoor localization, since it does not require dedicated infrastructure to be installed. The advent of deep learning has resulted in increasing interest in transfering its success to indoor localization [14,15,16,17,18]. Still, the nature of the fingerprinting method limits the accuracy that can ideally be reached, such that models are required that nevertheless provide a reliable space estimation while sacrificing as little expressiveness as possible. In this work, we proposed a new class of deep models, called DeepLocBox (DLB), that directly learns to estimate the area of the user by predicting a bounding box that contains its ground truth position. DLB consists of a dedicated output layer, a custom loss function, and a custom encoding scheme, whereas the body of the neural network remains interchangeable. During the evaluation on three distinct datasets, we showed that two neural network architectures equipped with the DLB head significantly outperformed the standard models with respect to success probability (up to 9.46%, 6.66%, and 3.86% increase). The DLB head can be adopted for a wide range of current deep model architectures for fingerprinting to provide more reliable space estimations and can be scaled towards the multi-building/multi-floor localization problem.

In future work, we will investigate its applicability for more complex architectures and examine whether the correlation between box size and error of the model can be further increased. In [18], we proposed a framework to provide adaptive area/space estimation in a setting where training data is continuously collected by crowdsourcing. We will explore the suitability of DLB in that setting, which would cancel the demand of for dedicated floor plan segmentation. Furthermore, we will adopt more complex output structures by extending our model towards polygon predictions.

## Figures and Tables

**Table 1:** Deep learning (DL) model parameters used during evaluation.

Model	Base				Head		
	HL	HU	Activation Fct.	Dropout Prob.	Output Layer	Output Activation Fct.	Loss Fct.
DNN	[1,2,3]	512	ReLU	0.5	2 units $\left(p_{x},p_{y}\right)$	linear	MSE loss
DNN–DLB					DLB output layer (Equation (8))	tanh	DLB loss fct (Equation (9))

**Table 2:** Convolutional neural network (CNN) model parameters used during evaluation.

Model	Base				Head	
	Layer 1	Layer 2	Layer 3	Layer 4	Output Layer	Loss Fct.
2D-CNN	16 × 16 Conv. + ReLU + 0.5 Dropout	16 × 16 Conv. + ReLU + 8 × 8 Maxpool + 0.5 Dropout	8 × 8 Conv. + ReLU + 4 × 4 Maxpool + 0.5 Dropout	Dense 128 + ReLU	2 units $\left(p_{x},p_{y}\right)$ + linear	MSE loss
2D-CNN-DLB					DLB output layer (Equation (8)) + tanh	DLB loss fct (Equation (9))

**Table 3:** Performance of DNN–DLB compared to space estimation models.

Dataset	Additional Aug. Training Data	Size-Gain (No Aug.) [m${}^{2}$]	Size-Gain (Aug.) [m${}^{2}$]	SUCC-Gain (No Aug.)	SUCC-Gain (Aug.)
RWTH (floor 4)	35.8%	−9.97	−9.54	9.46%	9.08%
Tampere (floor 1)	49.80%	−179.23	−207.33	5.48%	6.66%
UJI (bld. 0)	42.06%	−12.56	−103.74	3.71%	3.86%

**Table 4:** Comparison of center error of DLB and point error of DNN and 2D-CNN. The best performing models of both classes are compared.

Dataset	Model	Error [m]						
		Mean	Std	Min	25%	50%	75%	Max
RWTH Aachen (floor 4)	DNN (1 HL)	2.52	2.87	0.01	1.05	1.90	3.0	39.52
	DNN–DLB (1 HL)	2.04	3.53	0.0	0.65	1.25	2.37	63.38
	2D-CNN	5.31	4.45	0.01	2.23	4.15	7.2	37.38
	2D-CNN-DLB	2.36	3.4	0.01	0.84	1.54	2.9	60.11
Tampere (floor 1)	DNN (3 HL)	6.49	5.91	0.07	3.21	5.17	7.91	106.40
	DNN–DLB (1 HL, aug)	5.33	6.48	0.003	2.49	4.09	6.32	180.63
	2D-CNN	22.10	9.08	0.11	16.37	21.49	26.94	94.04
	2D-CNN-DLB	7.08	12.39	0.01	2.59	4.23	6.72	166.01
UJI (bld. 0)	DNN (3 HL)	6.89	5.90	0.02	2.96	5.32	9.16	79.63
	DNN–DLB (1 HL, aug)	6.31	5.91	0.01	2.63	4.75	8.09	121.32
	2D-CNN	12.38	7.82	0.05	6.65	10.75	16.52	60.47
	2D-CNN-DLB	6.76	6.93	0.04	2.84	4.94	8.06	111.66

**Table 5:** Multi-building/multi-floor performance comparison on the UJI dataset.

Info	Model	Building Success Rate	Floor Success Rate	Mean Error	Median Error
IPIN 2015 results [59] (multi-model)	MOSAIC	98.65%	93.86%	11.64 m	6.7 m
	HFTS	100%	96.25%	8.49 m	7.0 m
	RTLS@UM	100%	93.74%	6.20 m	4.6 m
	ICSL	100%	86.93%	7.67 m	5.9 m
Recent multi-model	CNNLoc [52]	100%	96.03%	11.78 m	-
Single DNN	Scalable DNN [50]	99.82%	91.27%	9.29 m	-
	DNN–DLB (best run)	99.64%	92.62%	9.07 m	6.32 m
	DNN–DLB (avg of 10 runs)	99.56%	92.12%	9.26 m	6.41 m

**Table 6:** Correlation between combinations of box size and center error of the boxes predicted by various models. The analysis was conducted for floor 0 of building 0 of the UJI dataset.

Model		Linear Regression					
**Label** **Aug.**	β **(Equation (6))**	**Y**	**X**	$\beta_{0}$	$\beta_{1}$	**p-Value**	$R^{2}$
No	5.0	*e*	w·h	24.78	2.35	2.83 × 10^−28^	0.18
		$e_{x}$	*w*	5.45	0.28	6.83 × 10^−23^	0.14
		$e_{y}$	*h*	4.78	0.23	1.85 × 10^−23^	0.15
		$e_{y}$	*w*	5.64	0.26	1.56 × 10^−16^	0.10
		$e_{x}$	*h*	5.32	0.06	6.98 × 10^−3^	0.01
	10.0	*e*	w·h	105.85	6.37	1.34 × 10^−16^	0.10
		$e_{x}$	*w*	11.33	0.43	5.22 × 10^−15^	0.09
		$e_{y}$	*h*	9.50	0.32	6.50 × 10^−20^	0.13
		$e_{y}$	*w*	11.76	0.36	2.75 × 10^−9^	0.06
		$e_{x}$	*h*	10.33	0.06	5.69 × 10^−2^	0.01
	15.0	*e*	w·h	235.91	10.72	2.64 × 10^−14^	0.09
		$e_{x}$	*w*	16.46	0.60	2.38 × 10^−15^	0.10
		$e_{y}$	*h*	14.22	0.41	2.54 × 10^−16^	0.10
		$e_{y}$	*w*	17.41	0.38	3.93 × 10^−6^	0.03
		$e_{x}$	*h*	15.47	0.02	6.21 × 10^−1^	0.00
Yes	5.0	*e*	w·h	30.61	2.37	1.72 × 10^−24^	0.16
		$e_{x}$	*w*	6.10	0.27	8.83 × 10^−26^	0.17
		$e_{y}$	*h*	4.97	0.24	4.34 × 10^−22^	0.15
		$e_{y}$	*w*	6.50	0.26	3.28 × 10^−12^	0.08
		$e_{x}$	*h*	5.52	0.06	1.65 × 10^−3^	0.02
	10.0	*e*	w·h	114.41	6.16	8.49 × 10^−20^	0.13
		$e_{x}$	*w*	11.61	0.47	1.27 × 10^−25^	0.17
		$e_{y}$	*h*	9.57	0.33	6.72 × 10^−19^	0.12
		$e_{y}$	*w*	12.57	0.37	2.53 × 10^−8^	0.05
		$e_{x}$	*h*	10.36	0.07	7.91 × 10^−3^	0.01
	15.0	*e*	w·h	242.37	14.05	2.05 × 10^−19^	0.12
		$e_{x}$	*w*	16.57	0.85	2.05 × 10^−29^	0.19
		$e_{y}$	*h*	14.11	0.40	1.32 × 10^−14^	0.09
		$e_{y}$	*w*	18.92	0.49	2.13 × 10^−5^	0.03
		$e_{x}$	*h*	14.94	0.11	3.21 × 10^−3^	0.01

## Data Availability

An internal dataset was used during initial validation. The final evaluation was conducted on two public datasets that are available online.
